# RELATIVE FAT MASS PREDICTS SYMPTOMATIC KNEE OSTEOARTHRITIS IN OLDER ADULTS WITH METABOLIC SYNDROME: A LONGITUDINAL COHORT STUDY

**DOI:** 10.2340/jrm.v58.45354

**Published:** 2026-09-04

**Authors:** Lei HUANG, Yuhui WANG, Yuhao CHEN, Weilong YAO, Xianxu ZHANG, Shicheng LI, Yong SUN, Changlin ZHOU, Bin QIAN, Zhiqiang LUO, Pandeng WEI

**Affiliations:** 1 Department of Orthopaedics, Lanzhou University Second Hospital, Lanzhou Gansu; 2 Department of Orthopaedics, Jingtai County People’s Hospital, BaiYin Gansu, China; 3 Lanzhou University Second Hospital, Lanzhou, Gansu, China

**Keywords:** CHARLS, knee osteoarthritis, RFM, obesity, metabolic syndrome

## Abstract

**Background:**

Relative fat mass is a novel anthropometric indicator that outperforms body mass index in assessing obesity. Although obesity is a known risk factor for knee osteoarthritis, the prospective association between relative fat mass and symptomatic knee osteoarthritis in middle-aged and older adults with metabolic syndrome remains unclear.

**Methods:**

This prospective cohort study used CHARLS data, including 3,301 middle-aged and older metabolic syndrome patients free of knee osteoarthritis at baseline. Participants were grouped into relative fat mass quartiles. Incident symptomatic knee osteoarthritis was the primary outcome. Multivariable Cox models, restricted cubic spline analyses, subgroup analyses, and receiver operating characteristic curves were used to assess associations and predictive performance.

**Results:**

Knee osteoarthritis incidence increased across relative fat mass quartiles (Q1: 21%; Q4: 37%). In fully adjusted models, those in the highest relative fat mass quartile had a 50% higher knee osteoarthritis risk compared with the lowest quartile (HR = 1.50, 95% CI: 1.18–1.90), with a significant dose–response trend. Restricted cubic spline analysis indicated a non-linear association. The relationship was stronger among rural residents. Relative fat mass demonstrated better predictive ability than body mass index (AUC: 0.587 vs 0.532).

**Conclusions:**

Among Chinese middle-aged and older adults with metabolic syndrome, higher relative fat mass was independently and non-linearly associated with an increased risk of incident symptomatic knee osteoarthritis. Relative fat mass outperformed body mass index as a predictive measure, supporting its use as a simple and effective tool to identify high-risk individuals and guide targeted prevention strategies.

## LAY ABSTRACT

Body fat affects health. Relative fat mass – calculated from height and waist – estimates fat better than BMI. We studied 3,301 Chinese middle-aged/older adults with metabolic syndrome (high blood pressure, sugar, and lipids). Over time, those with higher relative fat mass had a 50% greater risk of developing symptomatic knee osteoarthritis, especially in rural areas. relative fat massoutperformed BMI in predicting this risk. Relative fat mass is a simple, lowcost tool to identify high-risk metabolic syndrome patients for early prevention.

Knee osteoarthritis (KOA) is a major public health issue characterized by degenerative changes in articular cartilage and osteophyte formation, leading to pain, functional impairment, and limitations in daily activities (1). Of particular concern is that, with the acceleration of global population ageing, the burden of KOA on healthcare systems worldwide is expected to increase continuously (2). As the world’s second most populous country, China faces an escalating prevalence of KOA due to its ageing population, posing a significant public health challenge (3). A 4-year longitudinal study reported a cumulative incidence of symptomatic KOA as high as 8.5% among Chinese older adults (4).Traditionally, obesity has been considered the predominant risk factor for KOA (5), with its pathological mechanism primarily attributed to excessive mechanical load on the knee joint, where increased bodyweight directly accelerates cartilage and meniscus wear (6). However, a growing body of evidence indicates that KOA is not driven by a single factor but results from a complex interplay of genetic susceptibility, biomechanical alterations, metabolic dysregulation, and systemic inflammation (7). Metabolic syndrome (MetS), a cluster of conditions centred on insulin resistance, has emerged as a key entry point for investigating the “metabolic pathway” of KOA (8). MetS encompasses obesity, atherogenic dyslipidaemia, impaired fasting glucose, and hypertension, whose co-occurrence establishes a systemic state of chronic low-grade inflammation (9, 10). Pro-inflammatory cytokines (e.g., TNF-α, IL-1β, IL-6) and adipokines can reach the joint via the circulation (11), disrupting chondrocyte homeostasis, promoting extracellular matrix degradation, and exacerbating synovial inflammation – thereby driving KOA independently of, or synergistically with, mechanical loading (12). Moreover, numerous epidemiological and clinical studies have demonstrated significant associations between MetS, its individual components, and both the risk and radiographic progression of KOA (13). Consequently, individuals with MetS are subject to a dual insult: increased mechanical load due to higher bodyweight and accelerated cartilage degradation due to persistent systemic inflammation (14). This makes the MetS population a critical high-risk group for KOA prevention and intervention. Accurate risk stratification within this high-risk population is therefore essential. Importantly, the metabolic milieu of MetS – characterized by insulin resistance, chronic low-grade inflammation, and dysregulated adipokine secretion – may amplify the detrimental effects of excess adiposity on joint health (15). This suggests that the association between obesity-related metrics and KOA risk could be particularly pronounced in MetS populations, with metabolic derangements acting synergistically with mechanical loading to accelerate cartilage degradation. Therefore, accurate quantification of adiposity in MetS patients – beyond simple BMI measurement – may be particularly valuable for identifying those at heightened KOA risk. For a long time, body mass index (BMI) has been widely used as the standard measure of obesity and metabolic risk (16). However, BMI is a crude overall metric that cannot distinguish between fat mass and lean mass, nor does it reflect fat distribution (17, 18). In this context, relative fat mass (RFM), an emerging measure calculated from height and waist circumference, has shown great potential (19). RFM provides a more precise and convenient estimate of whole-body fat proportion, particularly abdominal fat accumulation, which is closely linked to metabolic hazards (20). Some previous cross-sectional studies have demonstrated a significant positive association between RFM and osteoarthritis, with higher RFM levels correlating with increased OA risk (21). Additionally, RFM has been shown to outperform traditional obesity indices in predictive performance (22, 23).

Based on this evidence, we hypothesized that RFM could offer unique value beyond conventional BMI in identifying the risk of symptomatic KOA. Investigating the independent association between RFM and KOA risk may not only deepen our understanding of the pathophysiological mechanisms underlying “metabolic KOA” but also provide practical epidemiological evidence for early and precise risk stratification in the MetS population.

## METHODS

### Study population

This study utilized data from the China Health and Retirement Longitudinal Study (CHARLS), a large-scale, nationwide, prospective cohort study targeting Chinese adults aged 45 years and older. CHARLS employed a multistage, stratified, proportional-to-population-size probability sampling method. The baseline survey was conducted in 2011, successfully recruiting 17,705 community-dwelling participants from 150 county- level units across 28 provinces, ensuring high representativeness of the middle-aged and older Chinese population. The study protocol was approved by the Biomedical Ethics Review Committee of Peking University (Approval No.:IRB00001052-11015), and all participants provided written informed consent prior to enrolment. Following the baseline survey, participants were followed up every 2–3 years to collect longitudinal information on socioeconomic status, health status, healthcare utilization, and other relevant variables. This study was conducted and reported in accordance with the Strengthening the Reporting of Observational Studies in Epidemiology (STROBE) guidelines.

**Fig. 1:**
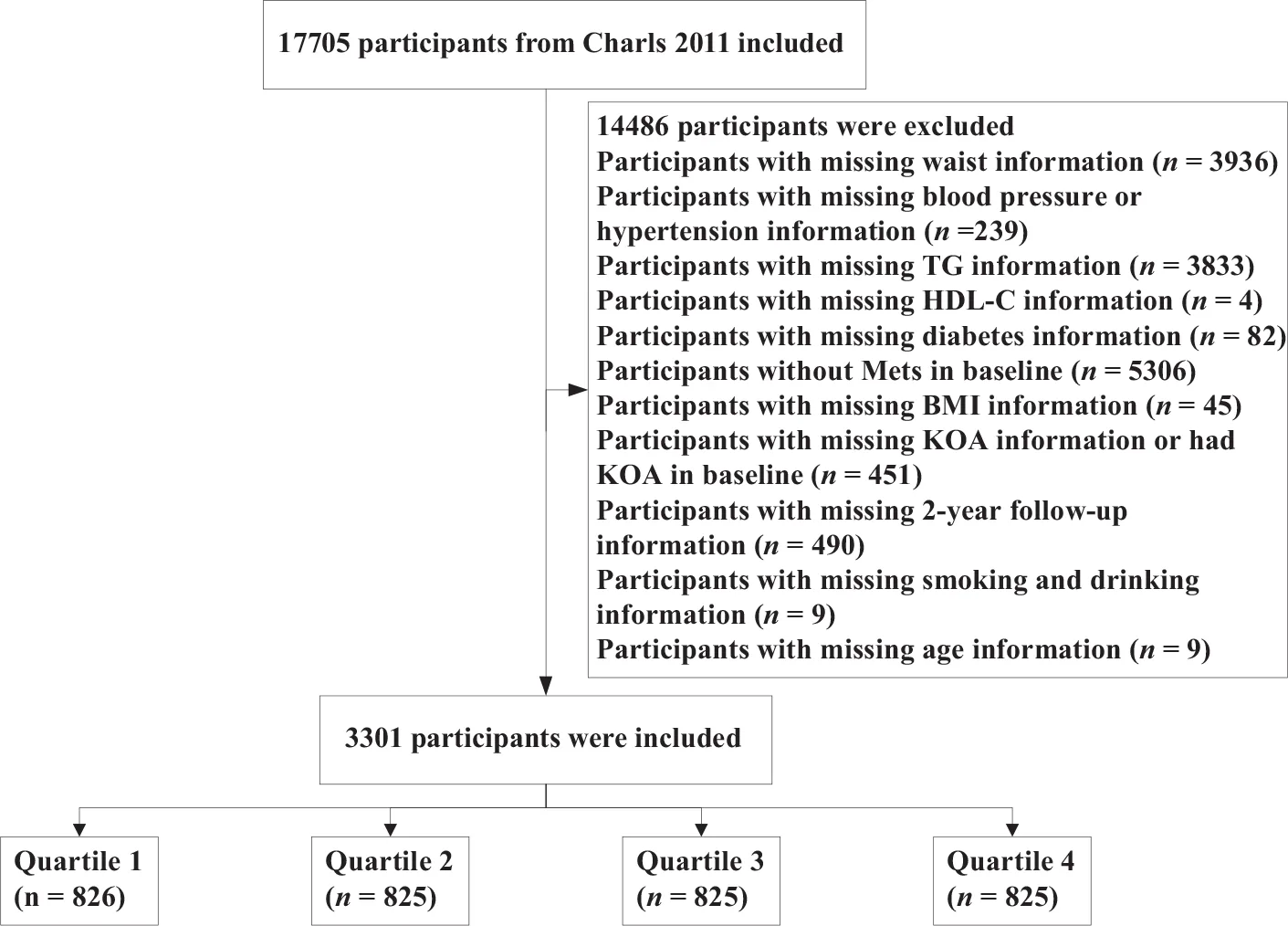
Flowchart for the inclusion and exclusion of participants.

The participant selection process is shown in Fig. 1. Initially, a total of 17,705 participants were included in this study. Participants were excluded based on the following criteria: missing waist circumference data (*n* = 3,936), missing blood pressure or hypertension information (*n* = 239), missing triglyceride (TG) data (*n* = 3,833), missing high-density lipoprotein cholesterol (HDL-C) data (*n* = 4), missing diabetes information (*n* = 82), absence of MetS at baseline (*n* = 5,306), missing BMI data (*n* = 45), missing KOA information or prevalent KOA at baseline (*n* = 451), missing 2-year follow-up data (*n* = 490), missing smoking or alcohol consumption information *(n* = 9), and missing age information (*n* = 9). After applying these exclusion criteria, a total of 3,301 participants were included in the final analysis. Based on baseline RFM values, all included participants were divided into 4 quartiles: Q1 (*n* = 828), Q2 (*n* = 828), Q3 (*n* = 826), and Q4 (*n* = 828). In addition, Table SI also gives detailed information on the ineligible participants and their differences from the included participants.

### Assessment of knee osteoarthritis

Symptomatic KOA in this study was determined using a combination of physical examination and questionnaire data from CHARLS. At baseline (2011) and at each subsequent follow-up, participants were asked: “Have you ever been diagnosed with arthritis or rheumatism by a doctor?” and “Do you often experience bodily pain?”. Participants who answered “yes” to both questions were then asked to indicate the specific site of pain on a body diagram. Symptomatic KOA was defined as meeting both of the following criteria simultaneously: self-reported physician-diagnosed arthritis/rheumatism, and pain located in either the left or right knee joint. To ensure that only incident cases were included, all participants were free of symptomatic KOA at baseline (2011). During follow-up waves (2013, 2015, 2018, and 2020), participants who met the above criteria for the first time were considered incident cases. This case definition is consistent with previous studies based on CHARLS data, ensuring comparability of results (24).

### Diagnosis of metabolic syndrome (MetS)

Adult MetS was diagnosed according to the criteria of the National Cholesterol Education Program Adult Treatment Panel III (NCEP-ATP III). Diagnosis required the presence of at least 3 of the following metabolic components: (1) waist circumference ≥ 90 cm for men or ≥ 80 cm for women; (2) serum triglycerides (TG) ≥ 150 mg/dL or current use of lipid-lowering therapy; (3) high-density lipoprotein cholesterol (HDL-C) < 40 mg/dL for men or < 50 mg/dL for women; (4) systolic blood pressure ≥ 130 mmHg or diastolic blood pressure ≥ 85 mmHg, or current antihypertensive treatment; and (5) fasting blood glucose ≥ 100 mg/dL or current treatment with insulin or oral hypoglycaemic agents.

### Calculation of relative fat mass (RFM)

RFM was calculated using the following formula: 64 − (20 × height (cm)/waist circumference (cm)) + (12 × sex), where sex is assigned a value of 1 for females and 0 for males (25).

### Covariates

Covariates included sociodemographic characteristics, anthropometric measurements, laboratory biomarkers, disease history and medication use, and health behaviours. Sociodemographic characteristics included age, marital status (married/unmarried), residence type (urban/rural), and educational attainment (less than primary, primary, junior high, senior high, or above). Anthropometric measurements included systolic blood pressure, diastolic blood pressure, waist circumference, BMI, and RFM. Laboratory biomarkers included haematological parameters (white blood cell count, mean corpuscular volume, platelets, haematocrit, haemoglobin), renal function indicators (blood urea nitrogen, creatinine, uric acid), lipid profile (total cholesterol, TG, HDL-C, low-density lipoprotein cholesterol [LDL-C]), fasting blood glucose, glycated haemoglobin, and C-reactive protein (CRP). Disease history was self-reported based on physician diagnoses and included hypertension, diabetes, lung disease, digestive disease, and dyslipidaemia; antihypertensive medication use was also recorded. Health behaviours included smoking status (never, former, current) and alcohol consumption status (never, former, current).

### Statistical analysis

All MetS participants were categorized into 4 groups (Q1–Q4) according to RFM quartiles. Continuous variables with normal distribution were presented as mean ± standard deviation (SD), and group differences were assessed using one-way analysis of variance (ANOVA); skewed continuous variables were presented as median (interquartile range, IQR) and compared using the Kruskal–Wallis H test. Categorical variables were expressed as counts (%) and compared using the χ^2^ test.

To prospectively assess the association between RFM and incident symptomatic KOA, univariable and multivariable Cox proportional hazards regression models were constructed to estimate hazard ratios (HR) and 95% confidence intervals (CI). Covariate adjustment was performed in three models: Model 1, unadjusted; Model 2, adjusted for age, residence, education level, marital status, smoking, and alcohol consumption; Model 3, further adjusted for hypertension, diabetes, BMI, TG, HDL-C, LDL-C, and CRP.

Restricted cubic spline (RCS) analysis was used to examine potential non-linear relationships between RFM and KOA risk, fitted on the basis of Model 3. Receiver operating characteristic (ROC) curves were employed to evaluate the predictive performance of RFM and BMI for incident symptomatic KOA, and the area under the curve (AUC) was calculated and compared. Subgroup analyses were conducted according to age, sex, BMI, residence, marital status, hypertension, diabetes, education level, smoking, and alcohol consumption, with likelihood ratio tests used to assess statistical significance of interactions. Variance inflation factors (VIF) for all covariates were < 5, indicating no significant multicollinearity. All statistical analyses were performed using R software (version 4.4.0; R Foundation for Statistical Computing, Vienna, Austria), and two-sided *p*-values < 0.05 were considered statistically significant.

## RESULTS

### Baseline characteristics of participants

Baseline characteristics of the population are presented in Table I. A total of 3,301 participants with metabolic syndrome were included in this study and divided into 4 groups (Q1–Q4) based on RFM levels. Comparison of baseline characteristics showed that, except for residence (*p* = 0.8) and triglycerides (TG; *p* > 0.05), all other variables differed significantly across RFM quartiles. During follow-up, the risk of symptomatic KOA increased progressively with higher RFM levels, with incidence rates of 21% in Q1 and 37% in the highest RFM quartile (Q4). The mean age of participants ranged approximately from 58 to 60 years.

**Table I:** Baseline characteristics by quartiles of RFM among individuals with metabolic syndrome

Characteristic	Q1 *n* = 826^1^	Q2 *n* = 825^1^	Q3 *n* = 825^1^	Q4 *n* = 825^1^	*p*-value^2^
Age, year	59 (53, 65)	58 (52, 65)	57 (49, 64)	60 (54, 66)	< 0.01
Body mass index (kg/m²)	23.4 (21.5, 25.1)	25.8 (22.3, 28.2)	24.5 (23.0, 25.9)	27.8 (26.0, 29.7)	< 0.01
Waist (cm)	86 (79, 91)	95 (80, 100)	88 (85, 90)	97 (94, 101)	< 0.01
Systolic blood pressure (mmHg)	133 (121, 145)	134 (121, 148)	132 (119, 147)	139 (125, 152)	< 0.01
Diastolic blood pressure (mmHg)	79 (71, 87)	79 (71, 87)	78 (70, 86)	80 (72, 88)	< 0.01
Relative fat mass	26 (23, 28)	32 (30, 36)	41 (40, 42)	45 (44, 46)	< 0.01
White blood count	6.20 (5.20, 7.50)	6.10 (5.10, 7.50)	6.01 (4.99, 7.20)	6.30 (5.20, 7.50)	< 0.01
Mean corpuscular volume (fl)	92 (88, 97)	91 (87, 96)	90 (86, 94)	90 (86, 94)	< 0.01
Platelets (10^9^/L)	203 (161, 253)	204 (158, 251)	215 (168, 267)	222 (174, 271)	< 0.01
Blood urea nitrogen (mg/dL)	16.0 (13.5, 19.1)	15.2 (12.6, 18.3)	14.2 (11.8, 16.7)	14.3 (12.0, 17.0)	< 0.01
Glu (mg/dL)	116 (105, 139)	111 (103, 131)	108 (101, 123)	109 (101, 124)	< 0.01
Serum creatinine (mg/dL)	0.86 (0.76, 0.99)	0.81 (0.69, 0.95)	0.69 (0.62, 0.77)	0.69 (0.61, 0.78)	< 0.01
Total cholesterol (mg/dL)	192 (165, 217)	194 (170, 220)	197 (174, 225)	202 (177, 231)	< 0.01
Triglycerides (mg/dL)	153 (92, 218)	154 (104, 222)	151 (105, 202)	151 (109, 219)	> 0.05
HDL-C (mg/dL)	40 (35, 51)	40 (33, 48)	44 (37, 51)	43 (36, 49)	< 0.01
LDL-C (mg/dL)	111 (87, 134)	115 (92, 139)	119 (96, 146)	122 (98, 150)	< 0.01
CRP (mg/dL)	1.19 (0.64, 2.49)	1.31 (0.70, 2.41)	1.18 (0.63, 2.37)	1.61 (0.94, 3.04)	< 0.01
HbA1c (%)	5.20 (4.90, 5.60)	5.30 (5.00, 5.60)	5.20 (4.90, 5.60)	5.30 (5.00, 5.70)	< 0.01
Uric acid (mg/dL)	4.97 (4.13, 5.92)	4.64 (3.73, 5.65)	4.05 (3.48, 4.74)	4.28 (3.64, 5.03)	< 0.01
Haematocrit (%)	44 (41, 48)	43 (39, 47)	40 (37, 43)	41 (38, 44)	< 0.01
Haemoglobin (g/dL)	15.20 (14.00, 16.50)	14.90 (13.50, 16.20)	13.70 (12.70, 14.70)	14.00 (13.00, 14.90)	< 0.01
Marital status					< 0.01
Yes	755 (91%)	756 (92%)	714 (87%)	707 (86%)	
No	71 (9%)	69 (8%)	111 (13%)	118 (14%)	
Hukou type					0.8
Urban hukou	332 (40%)	350 (42%)	340 (41%)	345 (42%)	
Rural hukou	494 (60%)	475 (58%)	485 (59%)	480 (58%)	
Education level					< 0.001
No completion of primary school	262 (32%)	298 (36%)	457 (55%)	531 (64%)	
Sishu/home school/elementary school	212 (26%)	209 (25%)	164 (20%)	146 (18%)	
Middle school	220 (27%)	208 (25%)	133 (16%)	117 (14%)	
High school and above	130 (16%)	110 (13%)	71 (8.6%)	31 (3.8%)	
Antihypertensive medication					< 0.01
Yes	178 (22%)	234 (28%)	209 (25%)	303 (37%)	
No	648 (78%)	591(72%)	616 (75%)	522 (37%)	
Hypertension					< 0.01
Yes	250 (30%)	320 (39%)	293 (36%)	391 (47%)	
No	576 (70%)	505 (61%)	532 (64%)	434 (53%)	
Diabetes					< 0.01
Yes	356 (43%)	285 (35%)	215 (26%)	220 (27%)	
No	470 (57%)	540 (65%)	610 (74%)	605 (73%)	
Lung disease					< 0.01
Yes	86 (11%)	70 (8.5%)	44 (5.3%)	63 (7.6%)	
No	740 (89%)	755 (91.5%)	781 (94.7%)	762 (92.4%)	
Dyslipidaemia					< 0.01
Yes	86 (11%)	153 (19%)	113 (14%)	161 (20%)	
No	740 (89%)	672 (81%)	712 (86%)	664 (80%)	
Digestive Diseases					< 0.05
Yes	146 (18%)	160 (19%)	187 (23%)	149 (18%)	
No	680 (72%)	665 (81%)	638 (77%)	676 (82%)	
Smoking status					< 0.01
Never	240 (29%)	441 (53%)	770 (93%)	756 (92%)	
Former	146 (18%)	133 (16%)	12 (1.5%)	21 (2.5%)	
Current	440 (53%)	251 (30%)	43 (5.2%)	48 (5.8%)	
Alcohol consumption					< 0.01
Never	257 (31%)	429 (52%)	697 (84%)	712 (86%)	
Former	133 (16%)	104 (13%)	56 (6.8%)	53 (6.4%)	
Current	436 (53%)	292 (35%)	72 (8.7%)	60 (7.3%)	
KOA-new					< 0.01
No	650 (79%)	621 (75%)	560 (68%)	521 (63%)	
Yes	176 (21%)	204 (25%)	265 (32%)	304 (37%)	

1 Median (Q1, Q3); *n* (%).

2 Kruskal–Wallis rank sum test; Pearson’s χ^2^ test.

Glu: fasting blood glucose; HDL-C: high-density lipoprotein cholesterol; LDL-C: low-density lipoprotein cholesterol; CRP: C-reactive protein; HbA1c: haemoglobin A1c.

As RFM increased, the proportions of current smokers and drinkers decreased significantly. Regarding metabolic indicators, systolic and diastolic blood pressure, waist circumference, BMI, total cholesterol, low-density lipoprotein cholesterol (LDL-C), and C-reactive protein (CRP) all showed upward trends, whereas high-density lipoprotein cholesterol (HDL-C) and serum creatinine exhibited variable changes (all *p* < 0.01). Notably, although waist circumference generally increased with RFM, the median values across quartiles did not follow a strictly monotonic pattern (86, 95, 88, and 97 cm). This is attributable to the sex-specific nature of the RFM formula, which incorporates both height and waist circumference; when sexes are pooled, the sex composition across RFM quartiles inevitably varies, and the non-monotonic trend in waist circumference reflects this methodological feature rather than a biological inconsistency. Overall, the highest RFM quartile (Q4) still exhibited the largest waist circumference (97 cm).

**Fig. 2:**
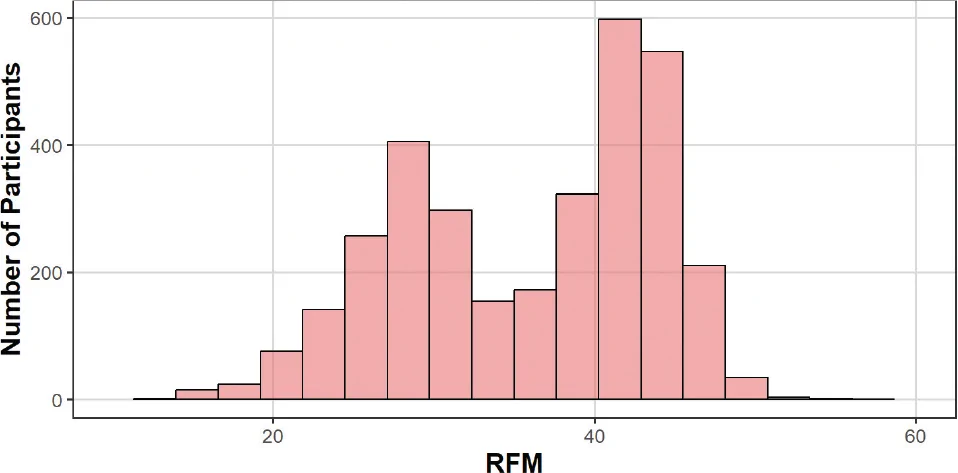
Distribution of RFM.

Additionally, the prevalence of hypertension and dyslipidaemia increased significantly in higher RFM groups (both *p* < 0.01), while the prevalence of diabetes showed a reverse trend. Educational attainment also differed significantly across groups, with the proportion of participants who had not completed primary school being markedly higher in the highest RFM quartile compared with Q1 (64% vs 32%, *p* < 0.001). Moreover, the use of antihypertensive medications increased progressively with RFM levels (22%→37%). Fig. 2 illustrates the distribution of RFM.

### Association between RFM and symptomatic KOA in MetS participants

As revealed in Table II, multivariable Cox proportional hazards models were used to evaluate the association between RFM and the risk of incident symptomatic KOA. When analysed as a continuous variable, each 1-unit increase in RFM was associated with a corresponding increase in KOA risk across Model I, Model II, and Model III.

**Table II:** Association between RFM and symptomatic knee osteoarthritis in individuals with metabolic syndrome

Exposure	Model I (HR, 95% CI)	*p*-value	Model II (HR, 95% CI)	*p*-value	Model III (HR, 95% CI)	*p*-value
RFM	1.02 (1.01, 1.02)	< 0.01	1.01 (1.00, 1.02)	< 0.01	1.01 (1.00, 1.01)	< 0.05
RFM quartile						
Q1	Reference		Reference		Reference	
Q2	1.16 (0.95, 1.42)	> 0.05	1.12 (0.92, 1.38)	> 0.05	1.09 (0.88, 1.35)	> 0.05
Q3	1.57 (1.29, 1.89)	< 0.01	1.43 (1.18, 1.75)	< 0.01	1.32 (1.04, 1.67)	< 0.05
Q4	1.86 (1.55, 2.24)	< 0.01	1.64 (1.35, 2.00)	< 0.01	1.50 (1.18, 1.90)	< 0.01
*P* for trend		< 0.01		< 0.01		< 0.01

MODEL I: crude model.

MODEL II: adjusted for Age, rural, education, marry, smoke, drink.

MODEL III: adjusted for age, rural, education, HBP, diabetes, marry, smoke, drink, BMI, TG, HDL, LDL, CRP.

To further elucidate the relationship between RFM and KOA incidence, participants were categorized into quartiles. Compared with the lowest quartile (Q1), in the fully adjusted Model III, the hazard ratio (HR) for KOA was 1.09 (95% CI:0.88–1.35) in Q2, indicating a non-significant increase in risk. The HRs for Q3 and Q4 were 1.32 (95% CI:1.04–1.67) and 1.50 (95% CI:1.18–1.90), respectively, suggesting that participants in Q3 and Q4 had a 32% and 50% higher risk of developing KOA compared with Q1 (*p* < 0.05). Trend analysis showed a significant dose–response relationship, with KOA risk increasing progressively across RFM quartiles (*p* for trend < 0.01).

### RCS

**Fig. 3:**
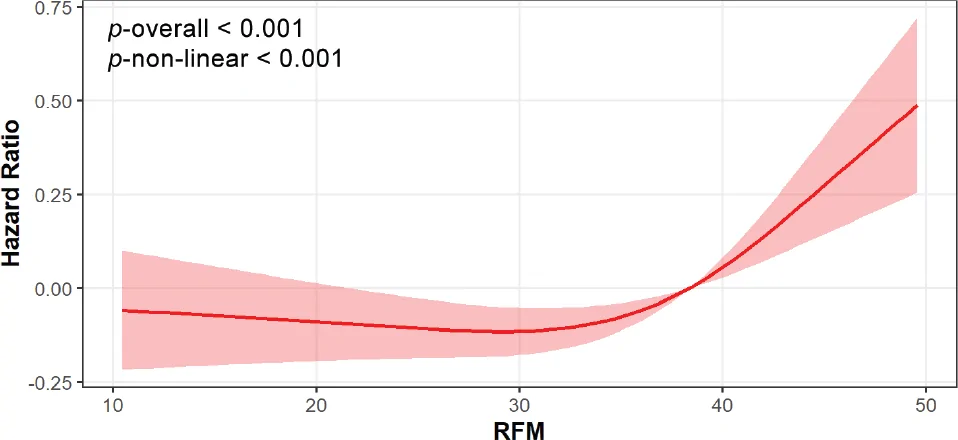
RCS analysis of RFM and symptomatic KOA in the MetS cohort. This model was adjusted for age, rural residence, education level, hypertension, diabetes, marital status, smoking, drinking, BMI, triglycerides, HDL, LDL, and CRP.

As shown in Fig. 3, to investigate whether the relationship between RFM and incident symptomatic KOA in MetS participants was non-linear, RCS analysis was performed based on Model III. The results demonstrated a significant non-linear association between RFM and the risk of symptomatic KOA (*p* for non-linearity < 0.001).To quantify this inflection point, we performed a piece-wise Cox regression with grid search, which identified a significant threshold effect at RFM = 28.95% (Table SII), above which the risk of symptomatic KOA increased substantially (HR = 1.035, 95% CI: 1.020–1.050, *p* < 0.001).

### Subgroup analyses

Table III presents subgroup analysis. It explores potential effect modification across participant characteristics. Statistical analysis indicated a significant effect modification by residence type (*p* for interaction = 0.046), with the association between RFM and KOA risk being notably stronger among rural residents (HR = 1.023) compared with urban residents. No significant interactions were observed for other subgroups, including age, BMI, sex, marital status, diabetes, alcohol consumption, and smoking (all *p* for interaction > 0.05), indicating that the positive association between RFM and KOA risk remained relatively consistent across these groups. After applying the Benjamini–Hochberg false discovery rate (FDR) correction for multiple comparisons, the subgroup-specific associations remained largely consistent; the corresponding FDR-adjusted q-values are presented in Table SIII.

**Table III:** Subgroup analyses of the association between the RFM level and KOA incidence in a population with MetS

Variable	HR (95% CI)	*p*-value	*p*-value for interaction
Age			0.557
< 60 years	1.019 (1.009, 1.029)	< 0.001	
≥ 60 years	1.014 (1.004, 1.025)	0.008	
BMI, kg/m²			0.360
< 28.5	1.014 (1.006, 1.022)	< 0.001	
≥ 28.5	1.023 (1.005, 1.041)	0.012	
Gender			0.478
Female	1.003 (0.995, 1.019)	0.44	
Male	0.999 (0.989, 1.008)	0.77	
Marital status			0.794
Yes	1.016 (1.009, 1.024)	< 0.001	
No	1.020 (0.996, 1.044)	0.11	
Hukou			0.046
Rural	1.023 (1.014, 1.034)	< 0.001	
Urban	1.009 (1.000, 1.019)	0.065	
Hypertension			0.065
Yes	1.026 (1.013, 1.039)	< 0.001	
No	1.011 (1.003, 1.020)	0.008	
Diabetes			0.816
Yes	1.018 (1.006, 1.030)	0.003	
No	1.016 (1.007, 1.025)	< 0.001	
Alcohol consumption			0.429
Never	1.011 (1.002, 1.019)	0.014	
Ever	1.003 (0.989, 1.019)	0.66	
Current	1.020 (1.001, 1.039)	0.04	
Smoking			0.780
Never	1.013 (1.004, 1.022)	0.005	
Ever	1.003 (0.976, 1.032)	0.81	
Current	1.009 (0.993, 1.025)	0.28	
Education			0.075
No completion of primary school	1.018 (1.007, 1.029)	0.001	
Sishu/home school/elementary school	1.022 (1.006, 1.039)	0.008	
Middle school	1.001 (0.989, 1.014)	0.87	
High school and above	1.001 (0.986, 1.015)	0.95	

This model was adjusted for age, rural residence, education level, hypertension, diabetes, marital status, smoking, drinking, BMI, triglycerides, HDL, LDL, and CRP.

### ROC

**Fig. 4:**
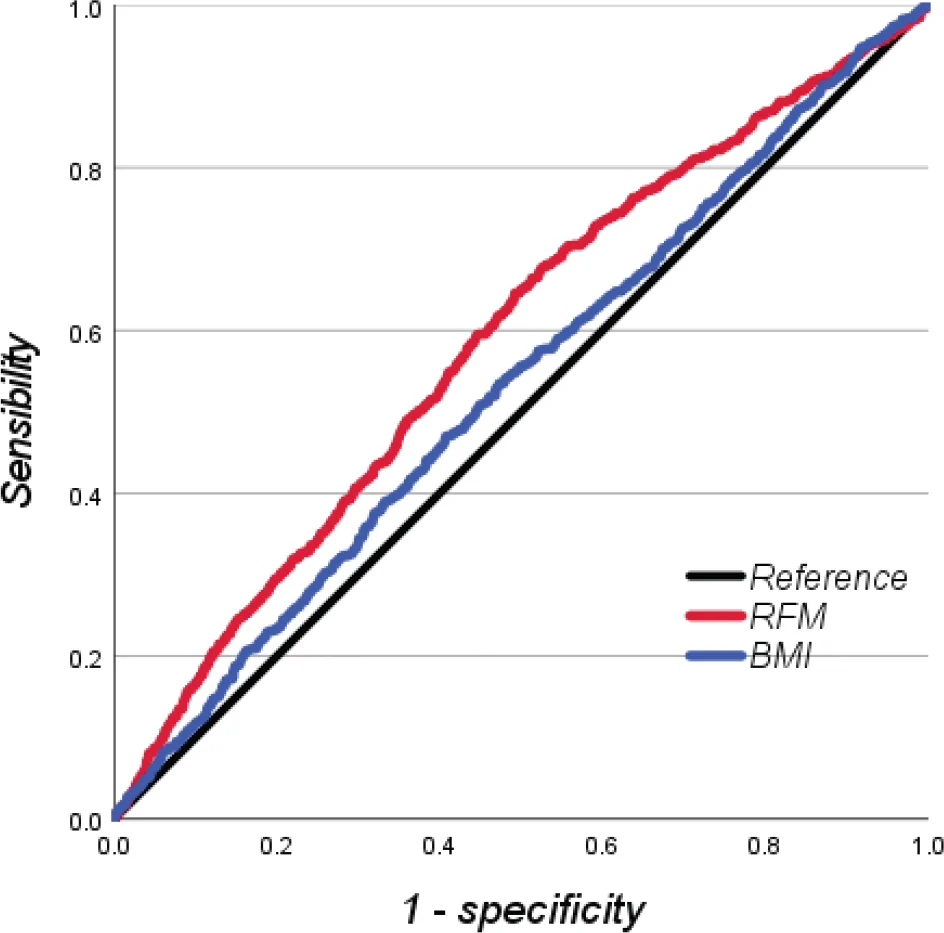
ROC curves of BMI and RFM for predicting incident symptomatic KOA in the MetS cohort.

ROC curve analysis seen in Fig. 4 was performed to evaluate the predictive performance of RFM and BMI for incident symptomatic KOA among MetS participants. Further analysis (Table IV) indicates that, during follow-up, the area under the curve (AUC) for RFM was 0.587, which was significantly higher than that for BMI (0.532). These results indicate that RFM can identify high-risk individuals for symptomatic KOA earlier and more accurately than BMI in MetS patients, providing a basis for timely targeted preventive and interventional strategies in clinical practice.

**Table IV:** Diagnostic performance of RFM and BMI for KOA

Characteristic	Cut-off	Sensitivity	Specificity	Youden index	AUC	*p*-value	95% CI	
							Lower	Upper
RFM	37.0	64.6%	50.8%	1.154	0.587	< 0.001	0.566	0.609
BMI	25.9	47.0%	59.3%	1.063	0.532	0.004	0.510	0.554

## DISCUSSION

In this first prospective cohort study of MetS individuals, RFM was significantly and positively associated with incident symptomatic KOA, with a non-linear dose–response relationship. Residence type significantly modified this association. The association was consistent across age, sex, and BMI, and RFM outperformed BMI by ROC analysis. These findings support RFM as a reliable KOA risk biomarker for early identification and precision prevention in MetS patients.

Obesity is an independent risk factor for KOA, not only by increasing mechanical load on the knee, which accelerates cartilage wear and structural joint changes, but also through systemic inflammatory pathways (26). Adipose tissue secretes pro-inflammatory cytokines, such as TNF-α and IL-6, which exacerbate cartilage degradation and synovial inflammation (27, 28). However, traditional obesity indices, such as BMI, have notable limitations: they cannot distinguish fat mass from lean mass, nor do they capture visceral fat accumulation (29). This may lead to underestimation or misclassification of metabolic risk, particularly in MetS patients, in whom abnormal visceral fat distribution is closely linked to insulin resistance and chronic inflammation. In contrast, RFM, calculated from height and waist circumference, provides a more accurate measure of body fat percentage and central obesity (30). Its advantages include simplicity, independence from muscle mass, and superior sensitivity and specificity in predicting obesity-related complications such as KOA, providing a strong rationale for focusing on RFM in MetS populations.

RFM, initially proposed by Woolcott et al., was designed to replace BMI for more precise estimation of whole-body fat percentage and to reduce misclassification of obesity across diverse populations (25). Emerging evidence has demonstrated its relevance in multiple chronic diseases, extending beyond simple body fat estimation (31). A large-scale study reported a significant positive association between RFM and stroke risk, with participants in the highest quartile exhibiting a 44% higher risk compared with the lowest quartile, and this association was significantly modified by age and BMI (19). In skeletal metabolism, a cross-sectional study found a significant negative correlation between RFM and lumbar bone mineral density (BMD), with a threshold effect (RFM = 20.5847), suggesting that excessive body fat may adversely affect bone health (32). Notably, in the context of arthritis, a study spanning populations in the United States and China revealed a complex non-linear relationship between RFM and arthritis risk. This study confirmed that RFM is an independent risk factor for arthritis in US men and women, with significant gender and population heterogeneity: among US women, arthritis risk decreased slightly when RFM was below 35.85 but increased sharply above this threshold, whereas no significant association was observed in Chinese men (33). Collectively, these studies establish RFM as a robust biomarker for predicting cardiovascular, skeletal, and joint disease risk.

Our findings not only confirm RFM as an independent risk factor for symptomatic KOA in MetS patients but also demonstrate its superior predictive value compared with traditional BMI, consistent with prior research. Our RCS analysis revealed a non-linear relationship between RFM and incident symptomatic KOA in MetS participants. This may reflect 2 distinct pathways. At low-to-moderate RFM, adipose tissue exerts limited pathogenic effects (mechanical cushioning, anti-inflammatory adiponectin). Above the threshold, excess adiposity increases joint loading and accelerates cartilage degradation (34). Hypertrophic adipose tissue also secretes pro-inflammatory adipokines, inducing systemic low-grade inflammation that damages cartilage and promotes synovitis. Our ROC analysis demonstrated a statistically significant but modest AUC improvement of RFM over BMI. This difference should not be overinterpreted for individual-level diagnosis, yet it is meaningful for population-based screening. In community settings where advanced imaging is unavailable, BMI remains limited by its inability to distinguish fat from lean mass (35). RFM, requiring only height and waist circumference, offers a practically cost-free, muscle-mass-independent upgrade. Applied to China’s vast MetS population, this modest AUC gain can translate into meaningful improvements in primary prevention risk stratification, as RFM permits a practical risk triage threshold derived from simple anthropometric measurements.

Furthermore, subgroup analyses further evaluated the consistency of the RFM–KOA association across different population strata. A nominal interaction was observed for residence type, with a stronger association among rural residents. Given the borderline significance level, this finding should be interpreted with caution and may reflect cumulative mechanical load from physical labour or limited healthcare accessibility in rural settings, although residual confounding cannot be fully excluded (36). Overall, the subgroup analyses did not reveal meaningful effect modification, and the primary finding of a positive RFM–KOA association remained robust across nearly all examined subgroups. The predictive advantage of RFM for KOA in MetS is mechanistically supported. RFM quantifies visceral fat, a core MetS feature, capturing the pathogenic obesity driving KOA. Key pathways include adipokine imbalance promoting cartilage degradation via JAK2/STAT3 and attenuating AMPK/NF‑κB protection (37, 38); FFA‑induced IKKβ/NF‑κB activation driving pro‑inflammatory cytokines and insulin resistance impairing chondrocytes (39–41); and saturated FFA triggering TLR4/NLRP3 inflammasome and IL‑1β/IL‑18 release (42, 43). Thus, RFM effectively identifies high‑risk individuals for targeted prevention.

This study has several strengths. It is the first prospective cohort to assess the RFM–KOA association in MetS patients, providing a simple early risk identification tool. The nationally representative large cohort had adequate sample size and follow-up. Multiple statistical methods and ROC analysis confirmed robust results and superior predictive performance of RFM over BMI. Limitations include residual confounding inherent to observational design, lack of longitudinal RFM data, potential measurement error, and limited generalizability beyond the Chinese elderly MetS population, warranting future multicentre validation.

## CONCLUSION

In this nationwide prospective cohort of Chinese MetS patients, higher RFM levels were independently and non-linearly associated with an increased risk of incident symptomatic KOA. RFM outperformed BMI as a predictive measure, suggesting its potential as a simple and effective tool to identify high-risk individuals for KOA in this high-risk population, thereby facilitating targeted preventive strategies.

## Supplementary Material


