# Supplementary material for: RELATIVE FAT MASS PREDICTS SYMPTOMATIC KNEE OSTEOARTHRITIS IN OLDER ADULTS WITH METABOLIC SYNDROME: A LONGITUDINAL COHORT STUDY
Source: J Rehabil Med. 2026 Sep 4;58:45354. doi: 10.2340/jrm.v58.45354 (PMC13548746; doi:10.2340/jrm.v58.45354)
Supplement: Supplementary file 1 [file JRM-58-45354-s1.pdf]

Supplementary Table SI. Comparison of baseline characteristics between included and excluded participants

Variable	Included	Excluded	P value
RFM, %	35 ± 12.92	30.29 ± 14.54	<0.001
Height, cm	1.58 ± 0.09	1.58 ± 0.09	0.307
Waist, cm	89.88 ± 11.74	82.51 ± 12.38	<0.001
BMI, kg/m^2	26.05 ± 42.37	23.55 ± 33.4	0.002
Age, year	59.07 ± 9.09	58.83 ± 10.41	0.189
WBC, $\times 10^9$/L	6.44 ± 1.93	6.18 ± 2.18	<0.001
BUN, mg/dl	15.46 ± 4.47	15.86 ± 4.71	<0.001
GLU, mg/dl	125.73 ± 48.4	104.19 ± 29.77	<0.001
SCR, µmol/L	0.79 ± 0.29	0.78 ± 0.21	0.419
TC, mg/dl	199.91 ± 41.73	190.22 ± 37.36	<0.001
TG, mg/dl	183.51 ± 143.43	115.7 ± 86.79	<0.001
HDL-C, mg/dl	43.84 ± 13.3	53.61 ± 15.2	<0.001
LDL-C, mg/dl	118.42 ± 38.75	115.04 ± 33.23	<0.001
CRP, mg/l	3.01 ± 7.2	2.72 ± 7.79	0.056
HA1bc, %	5.51 ± 1.09	5.16 ± 0.67	<0.001
UA, mg/dl	4.6 ± 1.29	4.41 ± 1.25	<0.001
HGB, g/dL	14.53 ± 2.26	14.33 ± 2.2	<0.001
**Gender**			<0.001
Female	1309 (39.7%)	7170 (49.8%)	
Male	1992 (60.3%)	7235 (50.2%)	
**Marital status**			0.002
No	369 (11.2%)	1889 (13.1%)	
Yes	2932 (88.8%)	12485 (86.9%)	
**Hukou type**			0.243
Urban hukou	1367 (41.4%)	5804 (40.3%)	
Rural hukou	1934 (58.6%)	8603 (59.7%)	
**Heart diseases**			<0.001
No	2819 (85.6%)	12518 (88.3%)	
Yes	474 (14.4%)	1656 (11.7%)	
**Stroke**			0.236
No	3195 (96.9%)	13845 (97.3%)	
Yes	102 (3.1%)	384 (2.7%)	
**Drinking status**			<0.001
Never	2095 (63.5%)	8238 (57.2%)	
Ever	346 (10.5%)	1481 (10.3%)	
Current	860 (26.1%)	4520 (31.4%)	
**Smoking status**			<0.001
Never	2207 (66.9%)	8412 (58.4%)	
Ever	312 (9.5%)	1114 (7.7%)	
Current	782 (23.7%)	4080 (28.3%)	
**Education level**			<0.001

No completion of primary school	1548 (46.9%)	6469 (45.1%)	
Sishu/home school/elementary school	731 (22.2%)	2996 (20.9%)	
Middle school	678 (20.6%)	2991 (20.8%)	
High school and above	342 (10.4%)	1891 (13.2%)	
**Hypertension**			<0.001
No	1134 (34.4%)	8721 (61.4%)	
Yes	2167 (65.6%)	5487 (38.6%)	
**Diabetes**			<0.001
No	2225 (67.4%)	13090 (92.6%)	
Yes	1076 (32.6%)	1039 (7.4%)	

Abbreviations: RFM, relative fat mass; BMI, body mass index; WBC, white blood cell count; BUN, blood urea nitrogen; GLU, glucose; SCR, serum creatinine; TC, total cholesterol; TG, triglycerides; HDL-C, high-density lipoprotein cholesterol; LDL-C, low-density lipoprotein cholesterol; CRP, C-reactive protein; HbA1c, glycated hemoglobin; UA, uric acid; HGB, hemoglobin.

Supplementary Table SII. Piece-wise Cox regression analysis of the association between RFM and symptomatic KOA risk

RFM range	HR	95% CI	P value
≤ 28.95%	0.994	0.988–1.001	0.064
> 28.95%	1.035	1.020–1.050	< 0.001

Footnotes: Threshold identified by grid search maximizing log-likelihood of piece-wise Cox model. Model adjusted for age, rural residence, education, hypertension, diabetes, marital status, smoking, drinking, BMI, triglycerides, HDL, LDL, and CRP. HR per 1% increase in RFM within each segment. P values from Wald tests.

Supplementary Table SIII. Subgroup analyses of the association between RFM and KOA incidence with FDR adjustment

Variable	HR (95% CI)	P	P for interaction	q-value*
**Age**			0.557	
<60	1.019 (1.009, 1.029)	<0.001		0.001
≥60	1.014 (1.004, 1.025)	0.008		0.019
**BMI**			0.360	
<28.5	1.014 (1.006, 1.022)	<0.001		0.002
≥28.5	1.023 (1.005, 1.041)	0.012		0.023
**Gender**			0.478	
Female	1.003 (0.995, 1.019)	0.44		0.558
Male	0.999 (0.989, 1.008)	0.77		0.877

**Marital status**			0.794	
Yes	1.016 (1.009, 1.024)	<0.001		0.002
No	1.020 (0.996, 1.044)	0.11		0.158
**Hukou**			0.046	
Rural	1.023 (1.014, 1.034)	<0.001		0.001
Urban	1.009 (1.000, 1.019)	0.065		0.097
**Hypertension**			0.065	
Yes	1.026 (1.013, 1.039)	<0.001		0.001
No	1.011 (1.003, 1.020)	0.008		0.018
**Diabetes**			0.816	
Yes	1.018 (1.006, 1.030)	0.003		0.01
No	1.016 (1.007, 1.025)	<0.001		0.002
**Alcohol consumption**			0.429	
Never	1.011 (1.002, 1.019)	0.014		0.025
Ever	1.003 (0.989, 1.019)	0.66		0.786
Current	1.020 (1.001, 1.039)	0.04		0.064
**Smoking**			0.780	
Never	1.013 (1.004, 1.022)	0.005		0.013
Ever	1.003 (0.976, 1.032)	0.81		0.881
Current	1.009 (0.993, 1.025)	0.28		0.368
**Education**			0.075	
No completion of primary school	1.018 (1.007, 1.029)	0.001		0.004
Sishu/home school/elementary school	1.022 (1.006, 1.039)	0.008		0.016
Middle school	1.001 (0.989, 1.014)	0.87		0.91
High school and above	1.001 (0.986, 1.015)	0.95		0.948

Footnotes: FDR-adjusted q-values were calculated using the Benjamini-Hochberg procedure across all subgroup-specific P-values. P-values <0.001 were set to 0.0009 for q-value computation.
